# CGRP antibody therapy in patients with drug resistant migraine and chronic daily headache: a real-world experience

**DOI:** 10.1186/s10194-021-01323-6

**Published:** 2021-09-20

**Authors:** Armin Scheffler, Hannah Schenk, Sebastian Wurthmann, Michael Nsaka, Christoph Kleinschnitz, Martin Glas, Dagny Holle

**Affiliations:** 1 Department of Neurology and Center for Translational Neuro- and Behavioral Sciences (C-TNBS), West German Headache Center, University Hospital Essen, University Duisburg-Essen, Hufelandstr. 55, 45147 Essen, Germany; 2 Department of Neurology and Center for Translational Neuro- and Behavioral Sciences (C-TNBS), Division of Clinical Neurooncology, University Hospital Essen, University Duisburg-Essen, Hufelandstr. 55, 45147 Essen, Germany

**Keywords:** Migraine, CGRP antibody, Therapy, Real-world, Chronic daily headache

## Abstract

**Background:**

Calcitonin gene-related peptide (CGRP) (receptor) antibodies (erenumab, fremanezumab and galcanezumab) are increasingly used in prophylactic treatment of migraine. In the approval studies, severely affected patients with migraine and chronic daily headache without any headache free days were excluded. Thus, less is known about the effectiveness of CGRP antibody treatment in this cohort.

**Methods:**

Clinical routine data of 32 patients with migraine and daily headache were analysed after three months of treatment with a CGRP antibody (16 erenumab, 7 galcanezumab, 9 fremanezumab), including changes of monthly headache days (MHD) monthly migraine days (MMD) and monthly acute medication intake (AMD) as well as migraine characteristics. Statistical analysis was performed with the Wilcoxon-Test. Migraine characteristics were analysed descriptively.

**Results:**

The number of MHD was significantly reduced (mean reduction (standard error), p-value): (-4.2 (1.3), *p* = 0.009) as well as MMD (-4.3 (1.6), *p* = 0.033). Four patients (13 %) reached a 50 % reduction regarding MHD and 8 patients (25 %) regarding MMD, migraine duration and intensity improved under therapy.

**Conclusions:**

Despite the low responder rate, CGRP antibodies can be effective at least in a few cases of severely affected patients with drug resistant migraine and chronic daily headache.

**Trial registration:**

Retrospective registered.

## Background

Monoclonal calcitonin gene-related peptide (CGRP) antibodies (in Europe erenumab, fremanezumab and galcanezumab) are being used more often in the prophylactic therapy of migraine. First real-world experiences and open label studies seem to confirm the benefit of the approval studies [1–3]. Nevertheless, all protocols of the approval studies excluded or had at least strong limitations regarding the severely affected patients with chronic daily headache (CDH) without any headache free days [4–6]. These patients were excluded from the trials primarily due to the expected poor response to therapy. Other studies showed a limited response to the pre-existing migraine prophylactic drugs regarding CDH [7, 8].

So far, no clinical data about the potential benefit of CGRP antibody therapy in this cohort are available. However, new therapeutic options are needed as these patients suffer more than others from migraine. We analysed the therapeutic effectiveness of CGRP antibodies in patients with migraine and daily headache after 3 months of treatment.

## Methods

We retrospectively analysed routine clinical data of 32 patients with migraine presented at the West German Headache Center, Department of Neurology, University Hospital Essen, Germany between November 2018 and July 2021. The analysis was approved by the independent ethics committee of the University Hospital Essen (19–9004-BO). All patients gave written consent to general analysis of their personal and clinical data. Patients meeting the following criteria: (a) Fulfilled criteria for migraine (ICHD-3) (b) CDH with headache every in day the last 90 days before treatment. (c) completion of a 90 days treatment interval with erenumab (16 patients, 70 mg/month), galcanezumab (7 patients) or fremanezumab (9 patients, 225 mg/month or 675 mg/3months)). Three patients had already received erenumab in the past. Patients treated with the three mAB were pooled for later statistical analysis. Clinical data reported by patients were compared to a paper-based or electronical headache diary. Monthly headache days (MHD) and monthly migraine days (MMD) were defined as the average monthly mean values over the respective total observation period of 90 days. A headache day was defined as a day with any kind of headache, a migraine day was defined by patients when they had severe pain, migraine pain characteristics (pulsating, one-sided pain), aura symptoms, vegetative symptoms like phono- or photophobia, nausea, vomiting, need for rest, or when triptans were taken. A 50 and 30 % responder rate were defined as a reduction by at least 50 or 30 % over the three months of treatment. Most patients answered questionnaires regarding different aspects of migraine: intensity of migraine (*n* = 32), duration of the migraine attack (*n* = 32), effect of acute therapy (*n* = 30), effect on the aura (*n* = 31), need for rest (*n* = 31), dizziness (*n *= 30), nausea (*n* = 31), phono- and photophobia (*n* = 31) as well as therapy satisfaction (*n* = 32). Data about changes of AMD was available of *n* = 30 patients. Due to reasons of reimbursement by the German statutory health insurance, all treated patients had tried six approved prophylactic drugs previously without sufficient treatment effects, had discontinued those due to side effects or were not eligible for intake due to contraindication. Approved drug classes were the following: betablockers (metoprolol or propranolol), tricyclic antidepressants (amitriptyline), calcium channel blockers (flunarizine), anticonvulsants (topiramate and valproic acid) and onabotulinumtoxin A. If there was a preexisiting migraine modulating comedication, medication was not altered. Data were analysed using SPSS software (IBM SPSS Statistics for Windows, Version 27.0. Armonk, NY, USA, IBM Corp), RStudio (Version 1.4, Boston, MA, USA, RStudio PBC) and Excel 2019 (Version 1809, Redmond, Washington, USA, Microsoft). Wilcoxon`s test was used to compare MHD, MMD and AMD before and after treatment. Bonferoni`s method for multiple comparisons was set (two-tailed *p*-value*3, alpha = 0.05). For statistical analysis of differences in response for MHD and MMD among the respective antibodies the Kruskal-Wallis-Test was used. Patient reported outcomes were analysed descriptively. (A part of the methods has already been used elsewhere [2]).

## Results

Clinical data of 32 patients with migraine and CDH were analysed. Details regarding demographic data, aura and duration of the disease, comedication and statistics about MMD, MHD and AMD before and after treatment are summarized in Table 1 (Table 1 here).

**Table 1:**
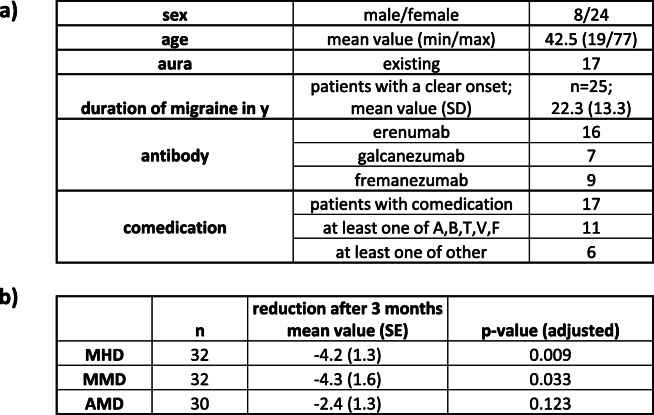
Patients´ characteristics and statistics. a) Patients characteristics, antibody therapy and comedication. A comedication of several medication was possible. Comedication: A: amitriptyline, B: betablocker, T: topiramate, V: valproic acid, F: flunarizine, others: magnesium and other antidepressive drugs like selective serotonine reuptake inhibitors). b) changes in monthly headache days (MHD) and monthly migraine days (MMD) and monthly acute medication intake (AMD) after 3 months of treatment after the respective CGRP antibody therapie. MHD and MMD were significantly reduced after 3 months of treatment

**Fig. 1:**
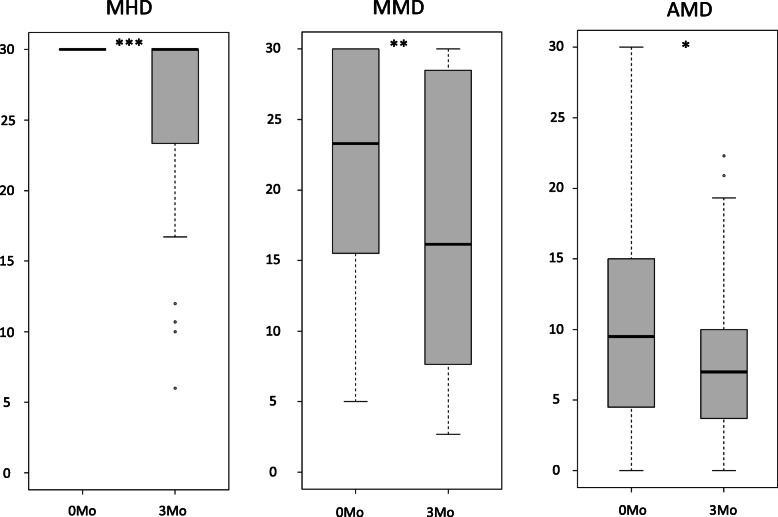
Treatment response. Boxplot of monthly headache days (MHD), monthly migraine days (MMD) and monthly acute medication intake (AMD) (y-axis) before treatment (0 Mo) and after 3 months (3Mo) of treatment (x-axis). Median, first and third qantile as well as minimum and maximum are shown. MHD and MMD were significant reduced, AMD alterations were not significant (*** *p* = 0.009; ** *p* = 0.033; * *p* = 0.123)

After 3 months of treatment a significant reduction of MHD and MMD was observed. Regarding medication overuse headache (MOH), 40 % (*n* = 12 of 30) before and 17 % (*n* = 5 of 30) after treatment had more than 10 acute medication intake days per month. However, no significant reduction of AMD was reached due to therapy after alpha adjustment (Fig. 1). Whereas 11 patients had any response to the treatment regarding daily headache, 21 patients showed no effect regarding MHD. 50 % responder rate of MHD was 13 % (*n* = 4) and 30 % responder rate was 22 % (*n* = 7). Regarding MMD, 50 % responder rate was 25 % (*n* = 8) and 30 % responder rate was 41 % (*n* = 13). There was no significant difference in the changes of MHD or MMD after three months between the three mAB (Kruskal-Wallis test: *p* = 0.767 and 0.813, respectively).

**Fig. 2:**
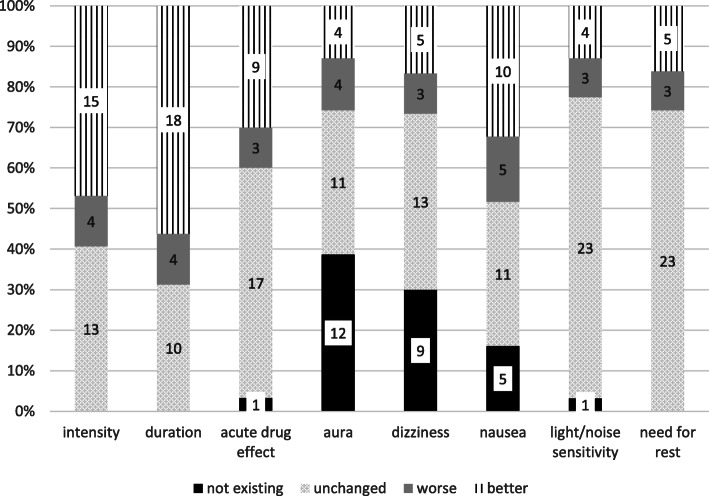
Migraine characteristics. Changes of migraine characteristics after three months of treatment. The relative proportion and the absolute number of patients who noticed the respective change are noted. All non-existent, non-applicable and non-occurring events are summarised under not existing

The improvement of migraine characteristics was reported, in particular an improvement of migraine intensity and duration, nausea as well as acute drug effect. No significant improvement was observed with regard to other clinical characteristics (Fig. 2).

Forty-one  % of patients (*n* = 13) were very satisfied or satisfied with the therapy, 44 % (*n* = 14) were moderately satisfied, 16 % (*n* = 5) were unsatisfied or very unsatisfied.

Side effects were reported by 38 % (*n* = 12) of patients, none of the patients reported a severe side effect. Main side effects were constipation with 21 % and nausea/vomiting with 16 % of all the reported side effects (*n* = 4 and *n* = 3, respectively).

## Discussion

Our real-world data shows the effectiveness and tolerability of CGRP antibody treatment in drug resistant patients with migraine and headache *every* day. Chronic daily headache (CDH) is often defined as the chronic form of headache diseases with at least 15 headache days per month [9]. Different mechanisms like acute medication overuse, neurotransmitter pathway modulations or alteration of pain related brain structures during a long burden of headache disease are discussed in pathophysiology of CDH [10].

The pivotal studies of the several CGRP antibodies focused on MMD reduction as the primary endpoint at different time points (50 % responder rate: galcanezumab: 27.6 % (120 mg/month) and 27.5 % (240 mg/month) [11]; erenumab 40 % (70 mg/month) and 41 % (140 mg/month) [4]; fremanezumab 38 % (675 mg/3 months) and 41 % (225 mg/month) [5]). Our study showed a low 50 % responder rate in MHD (13 %, *n* = 4) and a better response in MMD (25 %, *n* = 8). However, the pivotal studies did not include patients with daily headache. Little data are available for other approved licensed migraine prophylactic drugs used in CDH therapy and the existing data shows a limited effect. In a study focused on amitriptyline, the 50 % responder rate of MHD in a subgroup analysis of CDH (defined as more than 17 MHD) was 25 % after four weeks of treatment (*n* = 9 of 36) with significant results compared to placebo (*p* = 0.043), but no significant difference to placebo was observed after 8 weeks of treatment [7]. The treatment of patients with CDH (defined as headache *every* day) with onabotulinumtoxin A showed a 50 % MHD responder rate of 17.0 % (*n* = 18 of 106) from baseline at week 24 and 39.6 % (*n* = 19 of 48) at week 108. However, only less than half of the patients participated by week 108 (*n* = 48) compared to week 24 (*n* = 106), so the responder rate may be biased to the last timepoint [8].

Nevertheless, our data also shows a good response in MHD in at least these four patients and in 8 patients regarding MMD who previously failed all other first line therapies or were not able to receive them. Despite the low responder rate, patient reported better satisfaction than the actual reduction would lead on to expect. A possible reason is the observed improvement of the migraine characteristics, especially in duration and intensity of the migraine attack. Tolerability was good, and side effects were mild and comparable to previous CGRP mAb studies [1–3, 12].

There are some limitations of our study. First, patients who are affected with drug resistant migraine and CDH are rare and we decided to pool the data of all antibodies for statistical analysis. Thus, differences in the respective antibodies, e.g. better or worse effect cannot be identified. Nevertheless, considering the small number of cases for each antibody at least no significant difference between the respective antibody was detected. Furthermore, we have only retrospective real-world data and no placebo group. Great expectations in CGRP antibody therapy as a new and modern treatment option could feign an improvement. The long-term effect is not known. Further studies have to confirm the first impressions, that CGRP mAB could be beneficial to at least a few of these severely affected patients.

Although side effects were mild, one patient discontinued the therapy after 2 months of treatment because of symptoms of cold and allodynia of the scalp. Thus, the patient was not included to the analysis due to the incomplete treatment interval. In principle, a false positive therapy effect is therefore possible.

Another limitation is the differentiation between headache and migraine days in this special cohort. Patients with migraine and daily headache often suffer from migraine characterised pain and symptoms every day (e.g. phono- or photophobia, one sided headache) and only feel a worsening of the already existing symptoms in a migraine attack, making it difficult to distinguish between MHD and MMD. Thus, both parameters should be focused quantify the therapy effect. Especially headache-free days should be a target of the treatment of patients with CDH.

Regarding acute drug intake, 7 patients had no longer acute drug medication intake over 10 days a month after 3 months of treatment. This needs to be investigated in further studies, as due to the lack of significant change of AMD, this may be purely coincidental.

## Conclusions

Our data shows that CGRP antibodies could be a possible therapy option for at least a few severly affected patients who failed all previous therapies. Thus, a therapy trial may be useful, because a therapy response and an improvement of other migraine symptoms seems possible in this cohort. Further studies are needed to confirm long-term effects with a larger number of patients.

## Data Availability

The datasets used and/or analysed during the current study are available from the corresponding author on reasonable request.

## Abbreviations

AMD: monthly acute medication intake
CGRP: calcitonin gene-related peptide
CDH: chronic daily headache
ICHD-3: International headache classification
mAB: monoclonal antibody
MHD: monthly headache days
MMD: monthly migraine days
MOH: medication overuse headache
SE: standard error
